# Thinking About Forgetting: Awareness of Aging and Its Effect on Memory Lapses

**DOI:** 10.1093/geroni/igaf122.3238

**Published:** 2025-12-31

**Authors:** Ryan Fenstermacher, Taylor Nguyen

**Affiliations:** University of California, Irvine, Carlsbad, California, United States; University of California, Irvine, Irvine, California, United States

## Abstract

Awareness of aging (AoA) refers to a person’s perceptions of age-related changes across adulthood. Whereas some people may focus on perceived age-related gains with age, others may instead focus on age-related losses. These perceptions of aging are important for emotional well-being and physical health. The current study examines how AoA is related to one aspect of daily cognitive function, and how this association is related to daily stressors. We hypothesized that perceived age-related losses would be associated with increased memory lapses, an aspect of cognitive functioning that has been linked to later cognitive decline. Additionally, we explored whether daily stressors moderated this relationship. We used the Daily Diary Project and Daily Inventory of Stressful Events (DISE) from the third wave of the Midlife in the United States (MIDUS) study to examine the relationship between perceived age-related losses and memory lapses (N = 1,236). Multilevel models indicated that individuals who perceived greater age-related loss were more likely to experience memory lapses over a week, after adjusting for demographic factors and objective cognitive functioning. In addition, on days when they reported experiencing stressors, the positive relationship between perceived age-related loss and memory lapses was more pronounced compared to days without stressors. These findings suggest that one’s awareness of age-related loss may inform cognitive trajectories. Future research should examine longitudinal relationships between AoA, daily stresses, and long-term cognitive and physical outcomes.

